# Dietary Quercetin-3-Glucuronide Mitigates Oxidative Stress, Inflammation, and Fibroblast Transition by Regulating Nrf2 and Autophagy in Pulmonary Fibrosis

**DOI:** 10.1021/acs.jafc.5c13444

**Published:** 2026-01-14

**Authors:** Pei-Rong Yu, Chiao-Yun Tseng, Yu-Hsuan Liang, Yu-Ci Chang, Jing-Hsien Chen, Hui-Hsuan Lin

**Affiliations:** † Department of Nutrition, Chung Shan Medical University, Taichung City 40201, Taiwan; ‡ Department of Medical Laboratory and Biotechnology, Chung Shan Medical University, Taichung City 40201, Taiwan; § Clinical Laboratory, Chung Shan Medical University Hospital, Taichung City 40201, Taiwan

**Keywords:** pulmonary fibrosis, fibroblast-to-myofibroblast transition (FMT), quercetin-3-glucuronide (Q3G), nuclear factor erythroid 2−related factor 2 (Nrf2), autophagy

## Abstract

Pulmonary fibrosis involves oxidative stress, inflammation, and fibroblast-to-myofibroblast transition (FMT). Quercetin-3-glucuronide (Q3G) exhibits antioxidant and anti-inflammatory properties; however, its antifibrotic effects remain unclear. This study aimed to investigate the protective role of Q3G in epithelial injury-induced fibroblast activation, with a focus on nuclear factor erythroid 2–related factor 2 (Nrf2) and autophagy regulation. First, the decreased expressions of Nrf2 and an autophagy marker LC3 were detected in human emphysema compared with normal subjects. Further results demonstrated Q3G enhanced both coexpressions, while suppressing reactive oxygen species (ROS), interleukin (IL)-1β, IL-6, and extracellular matrix (ECM) deposition. The protective effects of Q3G were significantly reversed by Nrf2 silencing or autophagy inhibition. The Q3G-induced colocalization of Nrf2 and LC3 suggests a functional coupling between these pathways, which contributes to redox homeostasis and the attenuation of fibrosis. These findings indicate that Q3G mitigates oxidative stress, inflammation, and FMT via the coordinated activation of Nrf2 and autophagy, highlighting its therapeutic potential in pulmonary fibrosis.

## Graphical abstract

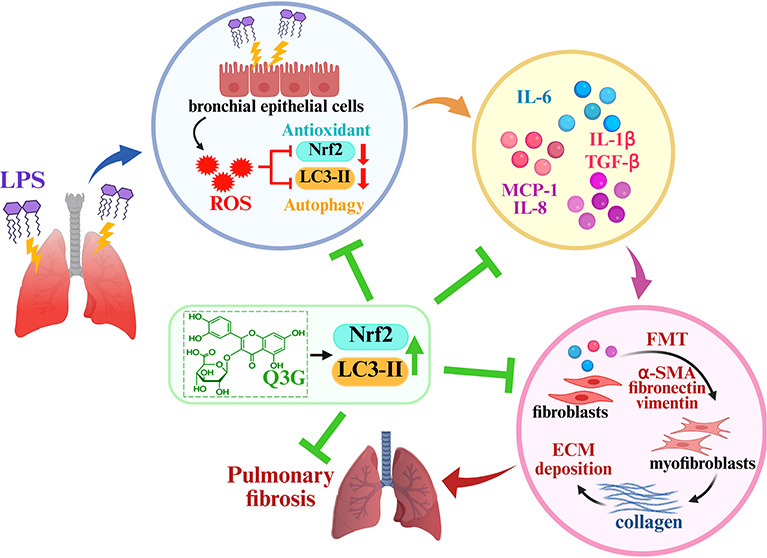

## 1 Introduction

Chronic obstructive pulmonary disease (COPD), encompassing conditions such as emphysema and chronic bronchitis, is defined by persistent airflow limitation, chronic airway inflammation, and progressive structural deterioration of lung tissue. This condition typically worsens over time and is associated with high morbidity and mortality worldwide.^1^ The pathophysiology of COPD involves a complex interaction of inflammation, oxidative stress, and tissue destruction within the airways and lung parenchyma. Inhalation of irritants leads to chronic inflammation in the airways, resulting in the recruitment of inflammatory cells and the release of cytokines. COPD is strongly linked to environmental and chemical exposures, including cigarette smoke, air pollutants, occupational irritants, as well as microbial components such as lipopolysaccharide (LPS), all of which trigger chronic airway inflammation and oxidative injury.^2^ The inflammatory response causes structural changes such as airway remodeling, mucous gland hyperplasia and fibrosis, leading to airflow obstruction.^2^ Furthermore, the destruction of lung parenchyma, particularly the alveolar walls, leads to emphysema, characterized by the enlargement of airspaces and loss of elastic recoil. The process impairs gas exchange and reduces the efficiency of breath.^3^

Oxidative stress is a key contributor to the pathogenesis of pulmonary fibrosis. Elevated levels of reactive oxygen species (ROS), generated from inflammatory cells, damaged epithelium, and environmental exposures, lead to redox imbalance and promote cellular injury. ROS can directly damage alveolar epithelial cells and activate redox-sensitive signaling pathways, such as nuclear factor kappa-light-chain-enhancer of activated B cells (NF-κB), resulting in increased production of pro-inflammatory and pro-fibrotic mediators.^4^ The fibrotic remodeling observed in pulmonary fibrosis is believed to stem from ongoing damage to bronchial and alveolar epithelium, which triggers the release of pro-inflammatory mediators and damage signals such as interleukin (IL)-1β, IL-6, tumor necrosis factor (TNF)-α, and particularly transforming growth factor (TGF)-β. These factors promote the activation and recruitment of immune cells, sustaining an inflammatory microenvironment that favors fibrogenesis.^5^ Among these cytokines, TGF-β is a critical driver of fibroblast-to-myofibroblast transition (FMT)—a process during which fibroblasts differentiate into highly contractile, extracellular matrix (ECM)-producing myofibroblasts marked by α-smooth muscle actin (α-SMA) expression. These myofibroblasts also display overproduction of ECM proteins such as collagen and fibronectin, along with increased expression of the mesenchymal marker vimentin. This transition is generally orchestrated by canonical Smad-dependent signaling. Continued activation of these molecular cascades enhances oxidative stress, promotes excessive ECM deposition, and leads to irreversible structural remodeling, ultimately contributing to the functional decline observed in fibrotic lung diseases^6^

Nuclear factor erythroid 2–related factor 2 (Nrf2) is a master regulator of cellular redox homeostasis and plays a vital role in protecting lung tissue against oxidative stress. Upon activation, Nrf2 translocates to the nucleus and upregulates a range of antioxidant and cytoprotective genes, including heme oxygenase-1 (HO-1), NAD­(P)­H quinone dehydrogenase 1 (NQO1), and glutathione-related enzymes. In the context of pulmonary fibrosis, Nrf2 signaling is often impaired, leading to redox imbalance, sustained inflammation, epithelial injury, fibroblast differentiation, and progression of fibrotic remodeling.^7^ In parallel, autophagy and oxidative stress regulation have emerged as critical cellular processes involved in the development and progression of pulmonary fibrosis. Microtubule-associated protein 1 light chain 3 (LC3), a key marker of autophagosome formation, reflects the dynamic activity of autophagy, which is essential for maintaining cellular homeostasis by removing damaged organelles and misfolded proteins. Dysregulated autophagy has been implicated in pulmonary fibrosis by facilitating fibroblast activation, excessive ECM deposition, and disrupted clearance of damaged proteins or organelles.^8^

Quercetin-3-glucuronide (Q3G) is a major circulating metabolite of quercetin, a dietary flavonoid extensively studied for its broad-spectrum bioactivities. Compared to its aglycone form, Q3G exhibits superior aqueous solubility, metabolic stability, and bioavailability, making it more suitable for *in vivo* applications. These properties contribute to its prolonged retention in circulation and enhanced cellular uptake.^9^ Also, Q3G show the effects of antioxidant, anti-inflammation, anticancer, neuroprotection. Owing to functional benefits, particularly in modulating redox homeostasis and inflammatory signaling pathways, Q3G has attracted increasing attention as a pharmacologically relevant compound with potential therapeutic utility in chronic inflammatory conditions.^10–12^ The biological effects of Q3G have been investigated in several disease models involving oxidative stress and immune dysregulation.^13,14^ Notably, Q3G has shown protective effects in LPS-induced lung injury model, primarily through the suppression of ROS generation and downregulation of pro-inflammatory mediators, leading to attenuate inflammation and pyroptosis.^15^ In the LPS-stimulated lung injury, Q3G also has been reported to activate Nrf2-mediated antioxidant pathways, thereby reducing oxidative stress. In parallel, the Q3G-activated Nrf2 may influence autophagy, which are essential for cellular homeostasis.^16^ However, the specific effects and underlying mechanisms of Q3G in pulmonary fibrosis have yet to be fully elucidated. Considering the central roles of oxidative stress and autophagy dysfunction in the pathogenesis of pulmonary fibrosis, it is plausible that Q3G may exert antifibrotic effects via these mechanisms. Therefore, the present study aims to investigate the protective effects of Q3G against epithelial injury-induced fibroblast activation, with a particular focus on its modulation of Nrf2 signaling and autophagy, and to elucidate its potential as a therapeutic candidate for pulmonary fibrosis.

## 2 Materials and Methods

### 2.1 In Vivo Experiment

The animal experiments were approved by the Institutional Animal Care and Use Committee (IACUC) of Chung Shan Medical University (IACUC approval number: 2642) and conducted in accordance with the ARRIVE guidelines. Six-week-old male C57BL/6 mice (body weight 20–22 g) were obtained from BioLASCO Co., Ltd. (Taipei, Taiwan) and housed in a controlled environment with a temperature of 25 ± 1 °C, with a relative humidity of 55 ± 5% and a 12-h light/dark cycle. After a one-week acclimation period, a total of 35 mice were randomly assigned to five experimental groups, as described in our previous study:^15^ (I) Control (*n* = 6), (II) lipopolysaccharide (LPS) challenge (*n* = 9), (III) LPS + quercetin-3-glucuronide (Q3G, 0.15 μmol/mouse; *n* = 9), (IV) LPS + dexamethasone (Dex, 1 mg/kg; *n* = 6), and (V) Q3G alone (*n* = 5). Except for groups I and V, which received PBS, groups II to IV were intranasally (i.n.) administered a mixture of 7 μg LPS (L4391; Sigma-Aldrich, St. Louis, MO) and 1.2 U porcine pancreatic elastase (E1250; Sigma-Aldrich, St. Louis, MO) in a total volume of 40 μL, once every 10 days for 30 days. The pulmonary injury model, combining LPS with elastase, is a well-established method for inducing pulmonary inflammation, emphysema, and fibrosis, which collectively mimic key pathological features of chronic obstructive pulmonary disease (COPD).^17^ In addition to the LPS challenge, mice in group III received Q3G (CAS No. 22688–79–5; purity >98.0%; ChemFaces, Wuhan, Hubei, China) by oral gavage at a dose of 0.15 μmol/mouse in 100 μL daily for 30 days. This dosage selection was based on our previous *in vivo* study,^15^ which demonstrated significant lung-protective effects of Q3G at this level without signs of systemic toxicity. Meanwhile, mice in group IV were intraperitoneally (i.p.) administered Dex, purchased from Sigma-Aldrich (St. Louis, MO), at a dose of 1 mg/kg once per week for four consecutive weeks. Due to its strong anti-inflammatory efficacy and immune-regulating function, Dex has been extensively utilized in the clinical management of lung-related inflammatory conditions.^18^ At the end of the 4-week treatment period, the mice were euthanized via cervical dislocation, following a previously described protocol,^19^ and lung tissues were immediately collected. The upper right lung lobes were fixed in 10% paraformaldehyde containing 10% (v/v) methanol for 24 h, followed by dehydration, paraffin embedding, and sectioning for histopathological analysis.

### 2.2 Histopathological Analysis

#### 2.2.1 Double Staining Immunohistochemistry (IHC)

The double staining kit (TADS03; BioTnA, Kaohsiung, Taiwan) was used with IHC technology to analyze both a lung disease tissue array (LUD481; Quickarrays, Inc., Fairfield, CA) and paraffin-embedded lung tissues from the animal experiments. The slides were deparaffinized with xylene and rehydrated through a graded ethanol series (100%, 95%, 80%, and 70% ethanol). Following sample preparation, the slides were stained with primary antibodies Nrf2 (AF0639; Affinity Biosciences, Cincinnati, OH) and LC3-II (NB100–2220; Novus Biologicals, Centennial, CO); or α-SMA (NBP2–33006; Novus Biologicals, Centennial, CO) and vimentin (sc-373717; Santa Cruz Biotechnology, Santa Cruz, CA), according to the instructions provided in the datasheet of the double staining kit. The positive area were quantified using ImageJ software.

#### 2.2.2 Masson’s Trichrome Staining

The paraffin-embedded lung tissues were sectioned at a thickness of 5 μm for the Masson’s trichrome staining, and the procedure was performed in accordance with a previous study.^20^ The process began with deparaffinization in xylene, followed by rehydration through a graded ethanol series (100%, 95%, 80%, and 70%). Slides were then treated with preheated Bouin’s solution (56 °C for 15 min) to enhance staining contrast, followed by washing in running tap water. Weigert’s iron hematoxylin was applied to stain the nuclei black, and the slides were then rinsed. Biebrich scarlet-acid fuchsin solution was used to stain the cytoplasm and muscle fibers red. This was followed by differentiation in phosphotungstic/phosphomolybdic acid solution to remove excess red stain from collagen. Aniline blue was then used to stain collagen fibers blue, followed by immersion in 1% acetic acid to fix the colors. After washing, the slides were dehydrated through graded ethanol, cleared in xylene, and mounted with a permanent mounting medium. In the final results, nuclei appeared black, muscle fibers red, and collagen blue. The collagen-positive area was quantified using ImageJ software.

### 2.3 In Vitro Experiment

#### 2.3.1 Cell Culture

The *in vitro* experiments utilized human lung fibroblast MRC-5 cells (Cat# 60023, RRID: CVCL_0440) obtained from the Bioresource Collection and Research Center (BCRC, Food Industry Research and Development Institute, Hsinchu, Taiwan). MRC-5 cells were cultured in Minimal Essential Medium with Earle’s salts (MEM), supplemented with 2.2 g/L sodium bicarbonate (NaHCO_3_), 10% fetal bovine serum (FBS), 1% l-glutamine, 1% sodium pyruvate, 1% nonessential amino acids, and 1% penicillin–streptomycin. Cells were maintained in a humidified incubator at 37 °C with 5% CO_2_.

#### 2.3.2 Cell Treatments

Based on our previous study with modifications,^15^ five treatment groups were established for the i*n vitro* experiments, as follows: (I) Control, (II) LPS stimulation, (III) LPS + Q3G, (IV) siNrf2 + LPS + Q3G, and (V) chloroquine (CQ) + LPS + Q3G. In group II, MRC-5 cells were exposed to LPS (10 μg/mL) for 24 h. In group III, cells were pretreated with Q3G (0.25 μM) for 6 h, followed by coincubation with LPS for an additional 24 h. For group IV, cells were transfected with small interfering RNA targeting Nrf2 (siNrf2, sc-37030; Santa Cruz Biotechnology, Santa Cruz, CA) according to the manufacturer’s instructions. For group V, cells were pretreated with the lysosomal inhibitor CQ (3 μM, CAS No. 50–63–5; Sigma-Aldrich, St. Louis, MO).^21^ Following Nrf2 knockdown in group IV and autophagy inhibition in group V, the cells were subsequently treated with Q3G for 6 h and then coincubated with LPS for 24 h, following the same protocol as group III.

#### 2.3.3 Transfection Analysis

Transient transfection was performed as previously described^16^ using the TransIT-X2 Dynamic Delivery System (Mirus Bio LLC, Madison, WI), a polymer-based transfection reagent. MRC-5 cells were transfected with Nrf2 siRNA (sc-37030), purchased from Santa Cruz Biotechnology (Santa Cruz, CA), in serum-free medium containing TransIT-X2 reagent for 12 h. Following transfection, the medium was replaced with complete medium containing double the concentration of FBS (20%), and cells were incubated for an additional 24 h.

#### 2.3.4 Conditioned Medium (CM) Model

To further investigate the lung-protective effects of Q3G on the pulmonary microenvironment, a coculture model using conditioned medium (CM) was established with modifications based on a previously described protocol,^22^ and applied in the experiments shown in Figures 4–6. Human bronchial epithelial BEAS-2B cells (Cat# iCell-h023; iCell Bioscience, Shanghai, China) were treated as described in section 2.3.2. The resulting CM was collected, filtered through a 0.22 μm membrane, and subsequently applied to MRC-5 cells for continuous treatment over a 96-h period. A schematic diagram of the CM model is presented in Figure 4A.

### 2.4 Western Blotting (WB)

The protein concentrations of the lung tissue homogenates were quantified using the Dual-Range BCA Protein Assay Kit (Energenesis Biomedical Co., Taipei, Taiwan). Based on previous study with modifications,^23^ 30–40 μg of purified protein was separated on an 8–15% SDS-PAGE gel. The separated proteins were then transferred onto nitrocellulose membranes (Millipore, Bedford, MA). To reduce nonspecific binding, the membranes were blocked with 5% fat-free milk at 4 °C for 1 h. The membranes were subsequently incubated overnight at 4 °C on a shaker with diluted primary antibodies, including Nrf2 (AF0639, Affinity Biosciences, Cincinnati, OH); LC3-II (NB100–2220) and α-SMA (NBP2–33006) from Novus Biologicals (Centennial, CO); IL-1β (sc-12742), collagen (sc-25974), PARP-1 (sc-56196), caspase-3 (sc-7148), IL-6 (sc-32296), TGF-β (sc-130348), fibronectin (sc-8422), and vimentin (sc-373717) from Santa Cruz Biotechnology (Santa Cruz, CA); p-Smad2 (3108t), Smad2 (5339t), p-Smad3 (9520t), and Smad3 (9523t) from Cell Signaling Technology (Danvers, MA), and β-actin (A5441) from Sigma-Aldrich (St. Louis, MO) as the internal control. The following day, the membranes were washed three times with tris-buffered saline containing 0.1% Tween-20 (TBST) for 10 min each at room temperature. Subsequently, the membranes were incubated with the diluted secondary antibodies antimouse IgG (A9044) and antirabbit IgG (A0545) from Sigma-Aldrich (St. Louis, MO) for 1 h at 4 °C with gentle shaking, followed by detection using enhanced chemiluminescence (ECL) reagent (Millipore, MA). Protein signals were then visualized with a luminescent image analyzer (ImageQuant LAS-4000; Fujifilm Corporation, Japan).

### 2.5 Pro-Inflammatory Cytokine Assay

Following treatment, the supernatants from cell cultures were harvested and subjected to IL-1β quantification using the ELISA MAX Deluxe Set Human IL-1β (Cat# 437015; BioLegend, San Diego, CA), in accordance with the manufacturer’s protocol.

### 2.6 Apoptosis Analysis

Cell apoptosis was evaluated using Annexin V and 7-amino-actinomycin D (7-AAD) double staining with the Annexin V and Dead Cell Kit (MCH100105; Cytek Biosciences, Fremont, CA). After treatment, MRC-5 cells were labeled following the manufacturer’s protocol and analyzed with the Muse Cell Analyzer (Cytek Biosciences, Fremont, CA).

### 2.7 Oxidative Stress Assay

To evaluate oxidative stress in treated cells, the reactive oxygen species (ROS) levels were assessed using the Muse Oxidative Stress Kit (MCH100111; Cytek Biosciences, Fremont, CA) and analyzed with the Muse Cell Analyzer (Cytek Biosciences, Fremont, CA), according to the manufacturer’s protocol. The results were expressed as the percentage of ROS-positive cells relative to the total cell population (M2%).

### 2.8 Multiplex Detection of Inflammatory Proteins

To assess the expression profiles of multiple inflammation-related cytokines, the Human Inflammation Antibody Array (ab134003; Abcam, Cambridge, U.K.), which detects 40 inflammatory targets, was used in accordance with the manufacturer’s protocol. In this study, protein lysates were prepared from CM-treated MRC-5 fibroblasts. Prior to detection, treated cells were lysed, and the resulting protein lysates were adjusted to a concentration of at least 1 mg/mL. It is recommended to use 200–250 μg of total protein per membrane for optimal detection. The membranes were imaged using a luminescent image analyzer (ImageQuant LAS-4000; Fujifilm Corporation, Japan), and signal intensities were quantified with Evolution-Capt software (Vilber, Paris, France). The levels of inflammatory factors were visualized as a heatmap, with red representing high expression and blue representing low expression.

### 2.9 Immunocytochemistry (ICC) Staining

The treated cells were rinsed and fixed in 4% paraformaldehyde at room temperature for an hour, followed by permeabilization with 0.1% Triton X-100 for an additional 15 min. To block nonspecific binding, the cells were incubated with 5% nonfat milk for 1 h. After blocking, the cells were incubated overnight at 4 °C with the target primary antibody. The following day, cells were incubated with the appropriate secondary antibody, and the nuclei were counterstained with 1 μg/mL 4′,6-diamidino-2-phenylindole (DAPI; Sigma-Aldrich, St. Louis, MO). Fluorescence images were acquired using an upright fluorescence microscope (ZEISS Axio Imager 2; Carl Zeiss, Oberkochen, Germany) and analyzed with ZEN Microscopy Software.

### 2.10 Statistical Analysis

All quantitative data are expressed as the mean ± standard deviation (SD) from at least three independent experiments. Statistical analyses were performed using SigmaPlot 10.0 (SYSTAT, San Jose, CA). A *p*-value of less than 0.05 was considered statistically significant.

## 3 Results

### 3.1 Coexpressions of Nrf2 and LC3-II in Emphysema Lungs of Human, and the Lung Tissues of the LPS-Induced Mice Treated with or without Q3G

It is widely recognized that emphysema represents a major pathological subtype of Chronic obstructive pulmonary disease (COPD), characterized by alveolar wall destruction and airspace enlargement.^1^ To explore the roles of Nrf2 and autophagy in COPD, human lung tissue arrays from normal lung and emphysematous lungs were subjected to double IHC staining with antibodies against Nrf2 and LC3-II (Figure 1A). As shown in Figure 1B,C, the expression levels of Nrf2 and LC3-II were significantly reduced in the emphysema group compared to the normal lung group. These findings suggest that downregulation of Nrf2 and LC3-II may be involved in the pathogenesis of COPD.

**Figure 1:**
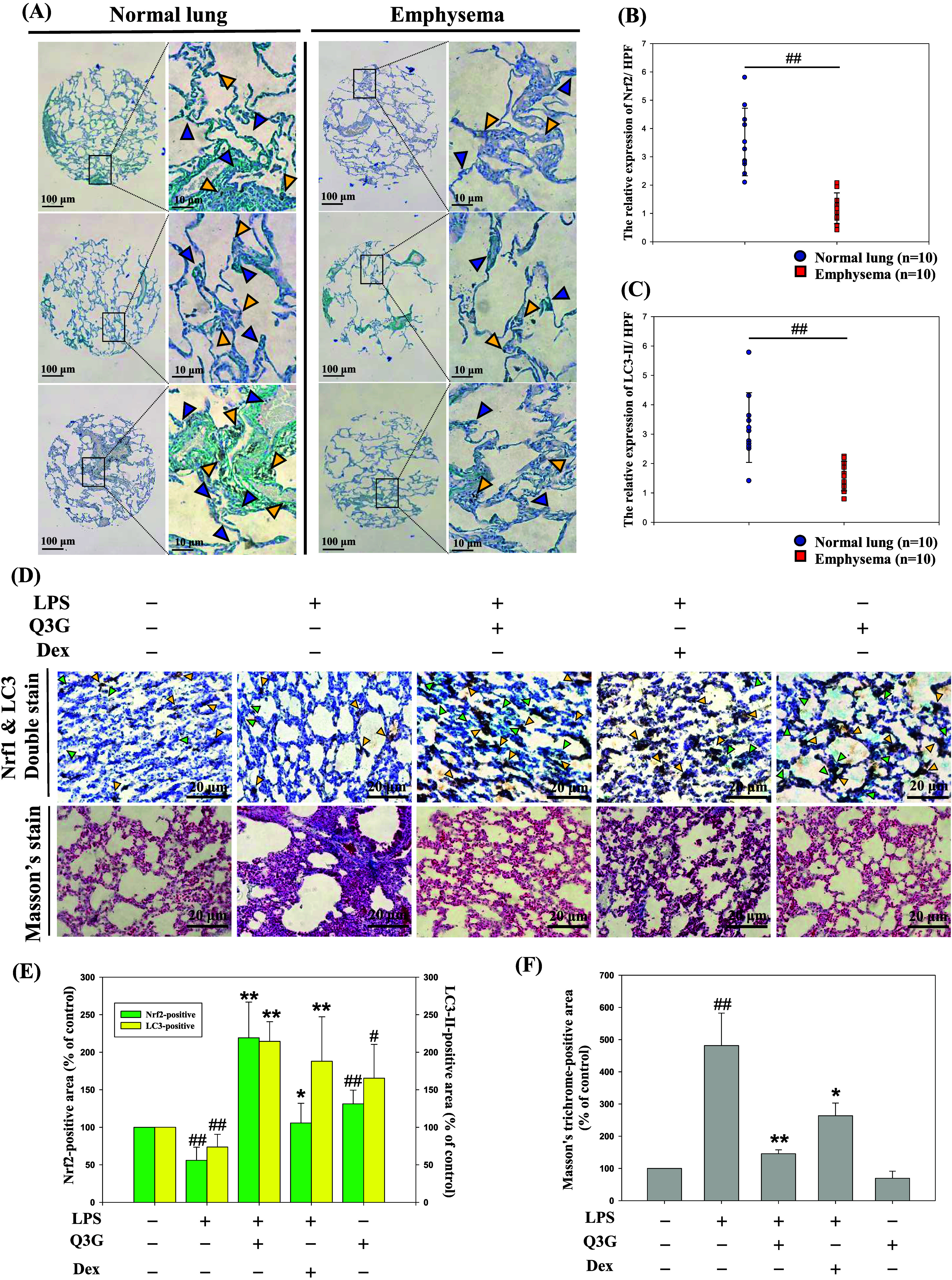
Coexpressions of Nrf2 and LC3-II in emphysema lungs of human, and the lung tissues of the LPS-induced mice treated with or without Q3G. (A) Representative images of human lung tissue arrays double-stained with Nrf2 and LC3-II antibodies to represent Nrf2 by the blue color and LC3-II by the brown color in normal and emphysema lungs. The relative expression of Nrf2 (B) and LC3-II (C) were measured in ten randomly selected fields from normal lung and emphysema, respectively. The quantitative data were presented are presented as mean ± SD (*n* = 10) from at least three independent experiments. ^##^ *p* < 0.01 compared with the normal lung group. (D) The C57BL/6 mice were treated with or without Q3G (0.15 μmol/mouse) for 30 days or Dex (1 mg/kg, i.p.) once weekly in the presence or absence of LPS once per 10 days. Representative images of lung sections from different treatments double-stained with Nrf2 and LC3-II antibodies (400×, *upper panel*) to represent the Nrf2 by the green arrows, and LC3-II by the yellow arrows, and Masson’s staining (400×, *lower panel*) to display the lung histological abnormalities. The Nrf2-positive area (*left axis*) and the LC3-II-positive area (*right axis*) (E), and Masson’s stain-positive area (F) are analyzed by image J. The quantitative data are presented as mean ± SD (*n* ≥ 3) from at least three independent experiments. ^#^ *p* < 0.05, ^##^ *p* < 0.01 compared with the control group. *p < 0.05, **p < 0.01 compared with the LPS group.

In subsequent *in vivo* experiments, a murine emphysema model was induced by intranasal instillation of lipopolysaccharide (LPS) and elastase, according to previously published protocols with modifications.^17^ Given that chronic inflammation is a key driver of lung injury and fibrosis,^2^ Dexamethasone (Dex), commonly used for the clinical management of lung inflammation,^18^ was included as a treatment group in the animal experiments. To investigate the effects of quercetin-3-glucuronide (Q3G) on Nrf2 and autophagy pathways, the paraffin-embedded lung sections were stained with Nrf2 and LC3-II (*upper panel*, Figure 1D). Compared to the control group, the LPS group showed a significant decrease in IHC-positive areas, whereas treatment with Q3G or Dex restored the expression levels suppressed by LPS (Figure 1E). Additionally, pulmonary fibrosis was assessed using Masson’s staining, with blue-stained areas indicating collagen deposition (*lower panel*, Figure 1D). Q3G and Dex markedly decreased the LPS-induced fibrotic lesion (Figure 1F).

### 3.2 Effects of Q3G on Fibrotic Factors in the Lung Tissues of the LPS-Induced Mice

To further evaluate the inhibitory effect of Q3G on lung fibrosis, markers associated with fibroblast-to-myofibroblast transformation (FMT) and fibrotic factors in lung tissues were examined. As shown in Figure 2A, double staining revealed that LPS challenge induced a substantial increase in the coexpression of α-SMA (yellow arrows) and vimentin (blue arrows), indicating the activation of FMT. However, treatment with Q3G markedly reduced the positive staining areas of these markers, suggesting that Q3G effectively suppresses LPS-induced myofibroblast differentiation (Figure 2B). The antifibrotic potential of Q3G was further confirmed by Western blot analysis. As shown in Figure 2C, the protein levels of TGF-β and Collagen were notably upregulated following LPS administration. Treatment with Q3G significantly attenuated the expression of these fibrotic factors, with an inhibitory effect comparable to the clinical treatment, Dex. These results demonstrate that Q3G mitigates pulmonary fibrosis by suppressing the FMT process and downregulating key fibrotic proteins in LPS-induced mice.

**Figure 2:**
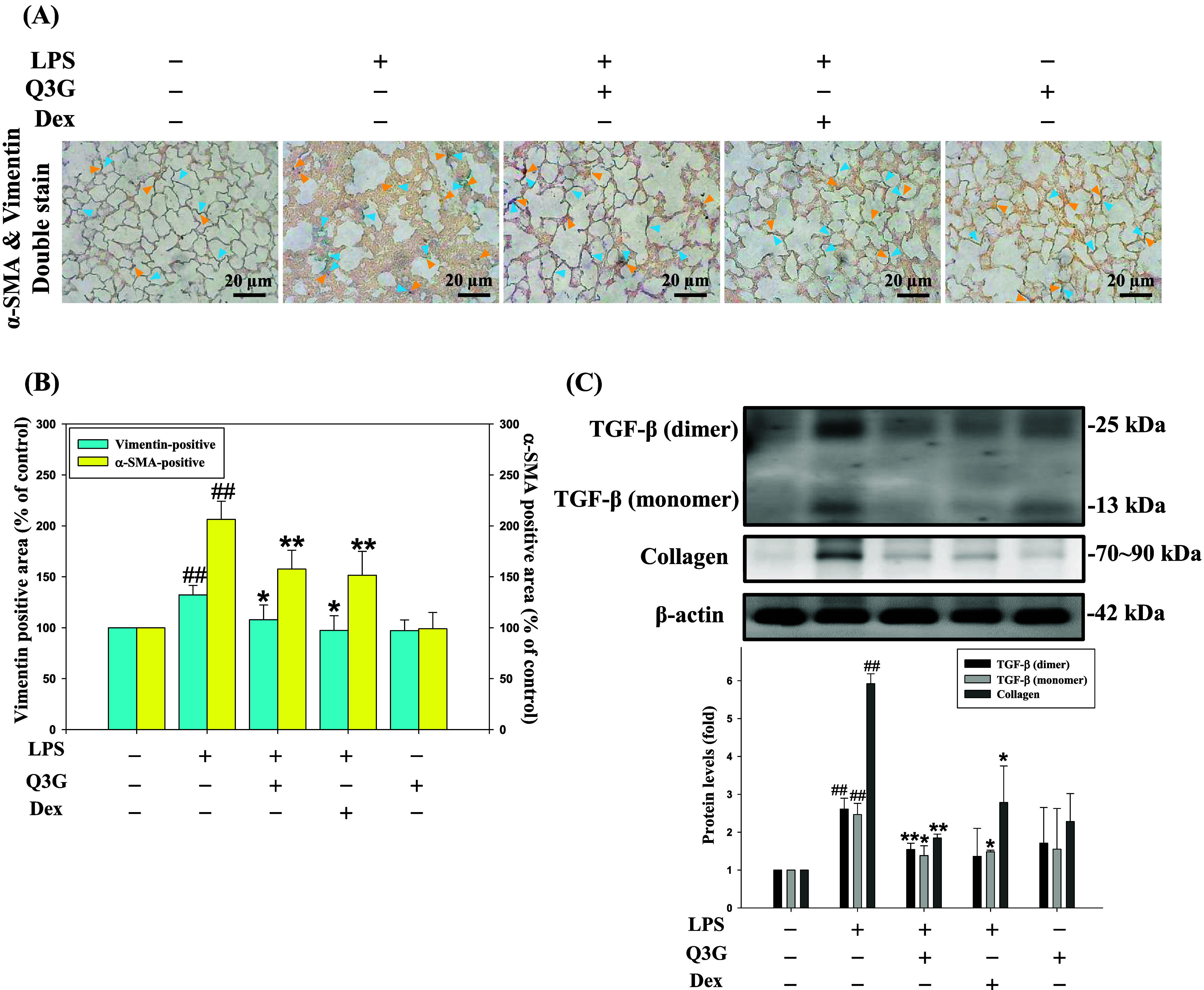
Effects of Q3G on fibrotic factors in the lung tissues of the LPS-induced mice. The C57BL/6 mice were treated with or without Q3G (0.15 μmol/mouse) for 30 days or Dex (1 mg/kg, i.p.) once weekly in the presence or absence of LPS once per 10 days. (A) Representative images of lung sections from different treatments double-stained with vimentin and α-SMA antibodies (400×) to represent vimentin by the blue arrows, and α-SMA by the yellow arrows (B) The vimentin-positive area (*left axis*) and the α-SMA-positive area (*right axis*) are analyzed by image J. The protein levels of TGF-β and collagen (C) were analyzed by Western blotting. β-actin was served as an internal control. The quantitative data are presented as mean ± SD (*n* ≥ 3) from at least three independent experiments. ^##^ *p* < 0.01 compared with the control group. *p < 0.05, **p < 0.01 compared with the LPS group.

### 3.3 Nrf2 and Autophagy Are Essential for the Q3G-Mediated Dysregulation of Inflammation and Fibrosis in the LPS-Induced MRC-5 Cells

To establish the role of Nrf2 and autophagy in the protective effects of Q3G, these pathways were first examined in human bronchial epithelial cells (BEAS-2B). As shown in Figure S1, LPS-induced downregulation of Nrf2 and mature IL-1β expression was reversed by Q3G treatment. Furthermore, Q3G-mediated upregulation of LC3-II was significantly enhanced by chloroquine (CQ). Notably, the inhibitory effects of Q3G on inflammatory factors were markedly abolished by either Nrf2 silencing (siNrf2) or autophagy inhibition with CQ (Figure S1), suggesting that Q3G’s anti-inflammatory properties are dependent on these two pathways in epithelial cells.

Next, human lung fibroblasts MRC-5 cells were employed in the *in vitro* experiments. To further investigate the underlying molecular mechanisms of Q3G-mediated Nrf2 and autophagy activation, Nrf2 siRNA transfection or treatment with the lysosomal inhibitor CQ were performed to clarify their respective roles. As shown in Figure 3A, Western blot analysis revealed that Q3G increased the expression of Nrf2, which was suppressed by LPS and abolished by siNrf2. In parallel, Q3G elevated the expression of LC3-II, and its accumulation was further enhanced by CQ treatment due to the inhibition of autophagosome degradation. These findings confirmed the effective knockdown of Nrf2 and autophagy inhibition by CQ. Additionally, LPS stimulation increased the levels of mature IL-1β and collagen, both of which were suppressed by Q3G treatment (Figure 3A). Consistently, ELISA results demonstrated that the Q3G-mediated reduction in LPS-induced IL-1β secretion was abolished by either Nrf2 knockdown or CQ treatment (Figure 3C). Notably, the anti-inflammatory and antifibrotic effects of Q3G were reversed under conditions of Nrf2 silencing or the lysosomal inhibitor, indicating that the protective effects of Q3G may depend on Nrf2 activation and intact autophagic flux (Figure 3A,C).

**Figure 3:**
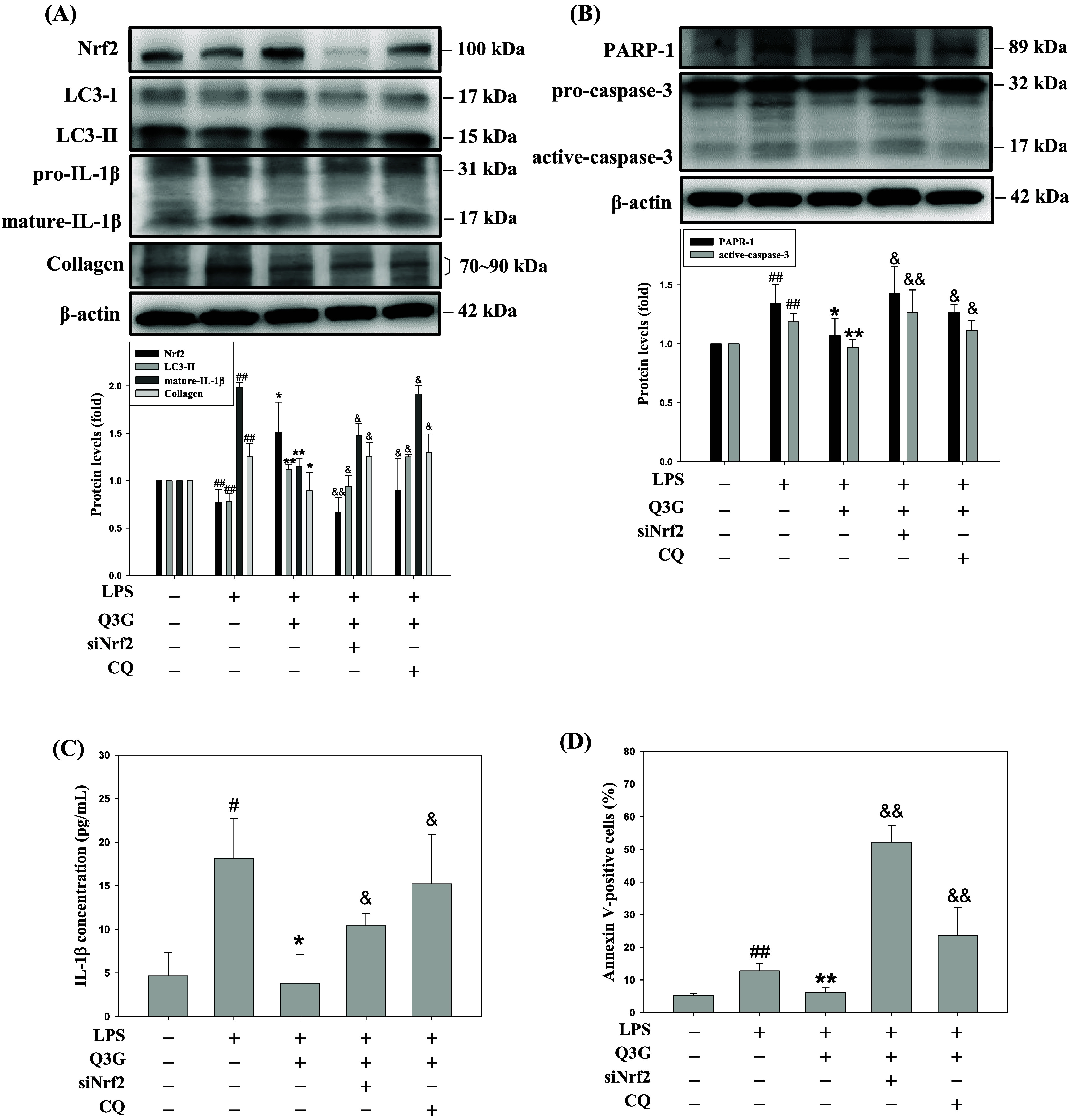
Nrf2 and autophagy are essential for the Q3G-mediated dysregulation of inflammation and fibrosis in the LPS-induced MRC-5 cells. MRC-5 cells were pretreated with or without Nrf2 siRNA for 12 h and CQ (3 μM) for 30 min, and then cotreated with Q3G (0.25 μM) for 6 h in the presence of LPS (10 μg/mL) for another 24 h. The protein levels of Nrf2, LC3-I/II, IL-1β, collagen (A) PARP-1 and caspase-3 (B) were analyzed by Western blotting. β-actin was served as an internal control. The IL-1β levels (C) in the culture medium of treatments cells were detected by ELISA assay. (D) The Annexin V-positive cells were presented as a number of total apoptotic cells stained with Annexin V-FITC and 7-AAD by Muse Cell Analyzer. The quantitative data are presented as mean ± SD (*n* ≥ 3) from at least three independent experiments. ^#^ *p* < 0.05, ^##^ *p* < 0.01 compared with the control group. *p < 0.05, **p < 0.01 compared with the LPS group. ^&^ *p* < 0.05, ^&&^ *p* < 0.01 compared with the LPS plus Q3G group.

Beyond inflammation and remodeling, dysregulated apoptosis of mesenchymal cells, such as fibroblasts, is a critical feature of chronic lung injury.^24^ To evaluate the roles of Q3G-modulated Nrf2 and autophagy in regulating apoptosis in LPS-stimulated MRC-5 cells, Western blot analysis and Annexin V/7-AAD staining were conducted. Western blot analysis showed an increase in the expression of PARP-1 and active caspase-3 in LPS-induced MRC-5 cells, whereas Q3G treatment significantly suppressed these apoptotic markers. This antiapoptotic effect was reversed by either Nrf2 knockdown or CQ treatment (Figure 3B). Similarly, Annexin V/7-AAD staining confirmed that Q3G reduced LPS-induced apoptosis, which was abolished when Nrf2 or autophagy was inhibited (Figure 3D). These results highlight that Q3G preserves fibroblast viability and stabilizes the lung microenvironment by mitigating early stage apoptosis through the mechanistic integration of Nrf2 signaling and autophagic flux, thereby preventing the subsequent pathological progression toward fibrotic remodeling.

### 3.4 Nrf2 and Autophagy Are Essential for the Q3G-Mediated Dysregulation of Oxidative Stress and Inflammation in the CM-Induced MRC-5 Cells

To characterize the profile of mediators released by injured epithelial cells, the levels of key pro-inflammatory and pro-fibrotic cytokines in the supernatant of LPS-treated BEAS-2B cells were quantified. As shown in Figure S2A–D, ELISA results demonstrated that LPS stimulation significantly increased the secretion of TGF-β, IL-1β, IL-6, and TNF-α compared to the control group. A heatmap further illustrated the distinct elevation of these cytokines in the LPS-induced conditioned medium (CM) (Figure S2E). These findings confirmed that LPS-challenged bronchial epithelial cells secrete a robust array of mediators capable of driving downstream fibrotic and inflammatory responses.

Based on these observations, a CM model was established to mimic the pathological mechanism of pulmonary fibrosis, in which injured bronchial epithelial cells generate oxidative stress and secrete pro-inflammatory mediators that influence adjacent fibroblasts.^25^ Human bronchial epithelial cells BEAS-2B were pretreated with or without Q3G, Nrf2 siRNA, or CQ, followed by LPS stimulation to induce epithelial damage. The CM was subsequently harvested and applied to human lung fibroblasts MRC-5 to investigate the downstream oxidative and inflammatory responses (Figure 4A). Prior to assessing these downstream responses, we first evaluated the impact of CM treatment on cell viability to exclude cytotoxic effects. As shown in Figure S3A, no overt morphological changes were observed in CM-treated MRC-5 cells under any condition. Flow cytometric analysis using the Cell Analyzer revealed that CM exposure did not significantly affect MRC-5 cell viability, as quantified in Figure S3B.

**Figure 4:**
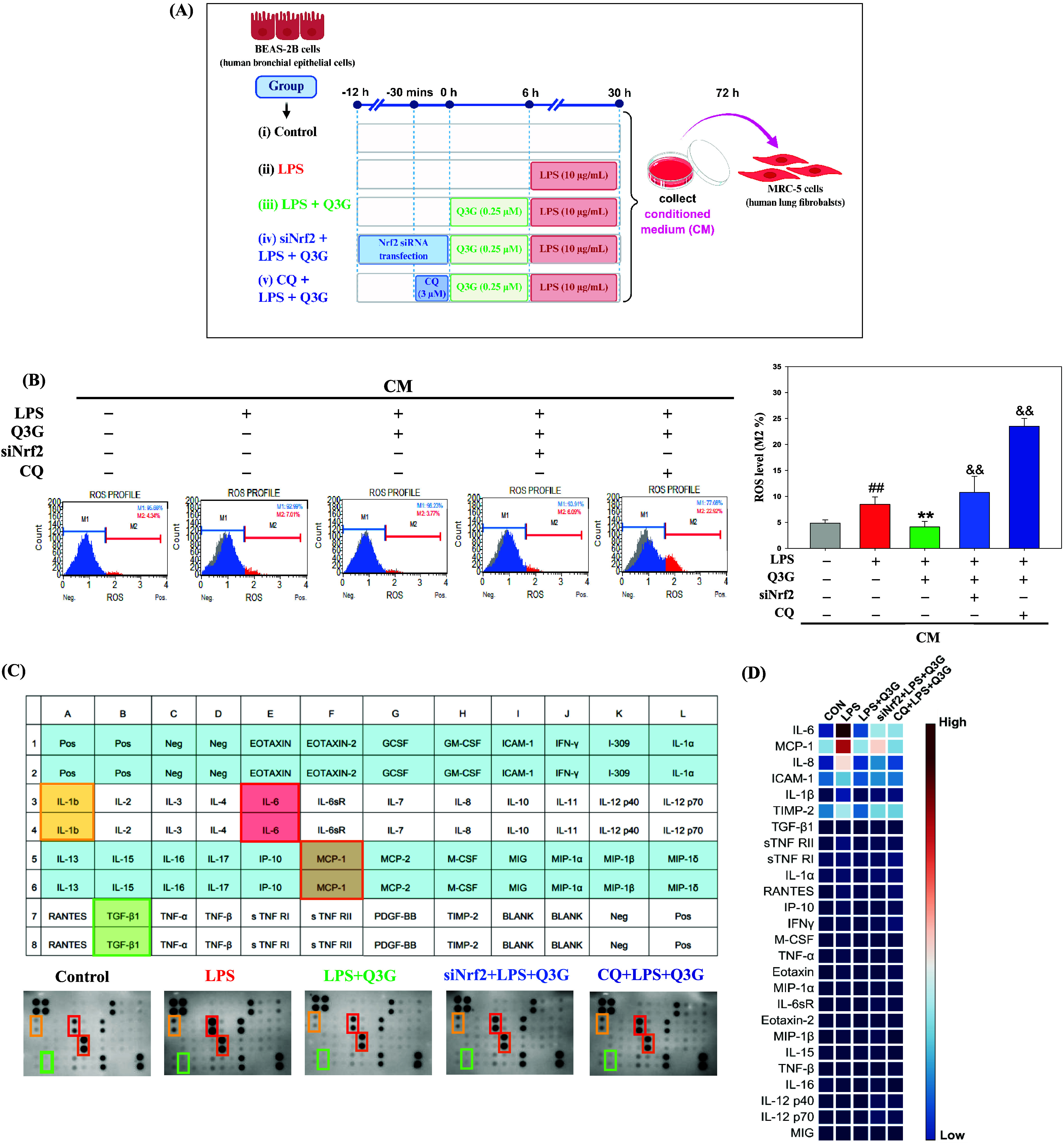
Nrf2 and autophagy are essential for the Q3G-mediated dysregulation of oxidative stress and inflammation in the CM-induced MRC-5 cells. (A) Experimental design for evaluating the effects of Q3G on LPS-induced epithelial injury and its influence on fibroblast activation via conditioned medium. BEAS-2B cells were pretreated with or without Nrf2 siRNA for 12 h and CQ (3 μM) for 30 min, and then cotreated with Q3G (0.25 μM) for 6 h in the presence of LPS (10 μg/mL) for another 24 h. Then the conditioned medium was collected and treated MRC-5 cells for 72 h. The schematic representation was created with BioRender.com. (B) The ROS were assayed by Oxidative stress kit with Muse Cell Analyzer (*left panel*). The quantitative data of ROS level are presented as mean ± SD (*n* ≥ 3) from at least three independent experiments. ^##^ *p* < 0.01 compared with the control group. **p < 0.01 compared with the LPS group. ^&&^ *p* < 0.01 compared with the LPS plus Q3G group. (C) The array map of the Human Inflammation Antibody assay illustrates the 40 targets on the Human Inflammation Antibody Array membrane (*upper panel*). Analysis of 40 human inflammatory factors in CM-induced MRC-5 cells utilizing the Human Inflammation Antibody Array (*lower panel*). (D) The levels of inflammatory factors were presented as a heatmap from high (red color) to low (blue color).

As shown in Figure 4B, treatment with LPS-induced CM significantly increased ROS generation in MRC-5 cells. Q3G treatment markedly suppressed intracellular ROS levels; however, this effect was significantly reversed when Nrf2 was silenced or autophagy was inhibited. In addition, a Human Inflammatory Cytokine Antibody Array was employed to assess cytokine profiles in MRC-5 cell lysates under different CM treatments (Figure 4C). The LPS-induced CM increased the expression of several inflammatory cytokines, including IL-6, IL-8, MCP-1, ICAM-1, and TGF-β, which were markedly reduced by Q3G treatment. However, this anti-inflammatory effect was weakened in the presence of Nrf2 silencing or CQ cotreatment. The corresponding heatmap further confirmed that Q3G suppressed LPS-induced levels of inflammatory cytokines in fibroblasts, and this suppression was dependent on both Nrf2 activity and autophagic function (Figure 4D). Collectively, these results indicate that Q3G ameliorates CM-induced oxidative stress and inflammation in lung fibroblasts, at least in part through Nrf2 activation and autophagy regulation.

### 3.5 Nrf2 and Autophagy Are Essential for the Q3G-Mediated FMT in the CM-Induced MRC-5 Cells

To investigate whether Q3G modulates FMT under the influence of epithelial-derived stimuli, immunocytochemistry (ICC) was first employed to examine the expression of α-SMA, a key marker of FMT. As shown in Figure 5A, immunofluorescence staining revealed a marked increase in α-SMA expression in MRC-5 cells treated with LPS-induced CM, indicative of myofibroblast differentiation. Q3G treatment significantly reduced α-SMA-positive staining, while this inhibitory effect was attenuated upon Nrf2 silencing or cotreatment with CQ (Figure 5B). In addition, given the prominent expression of IL-6 observed in the Inflammatory Cytokine Antibody Array (Figure 4D), the protein level of IL-6 in MRC-5 cells was further examined. As shown in Figure 5C,5D, IL-6 expression was markedly increased in the LPS-induced CM group and was significantly decreased by Q3G treatment. However, this suppressive effect of Q3G was diminished when Nrf2 was silenced or autophagy was blocked.

**Figure 5:**
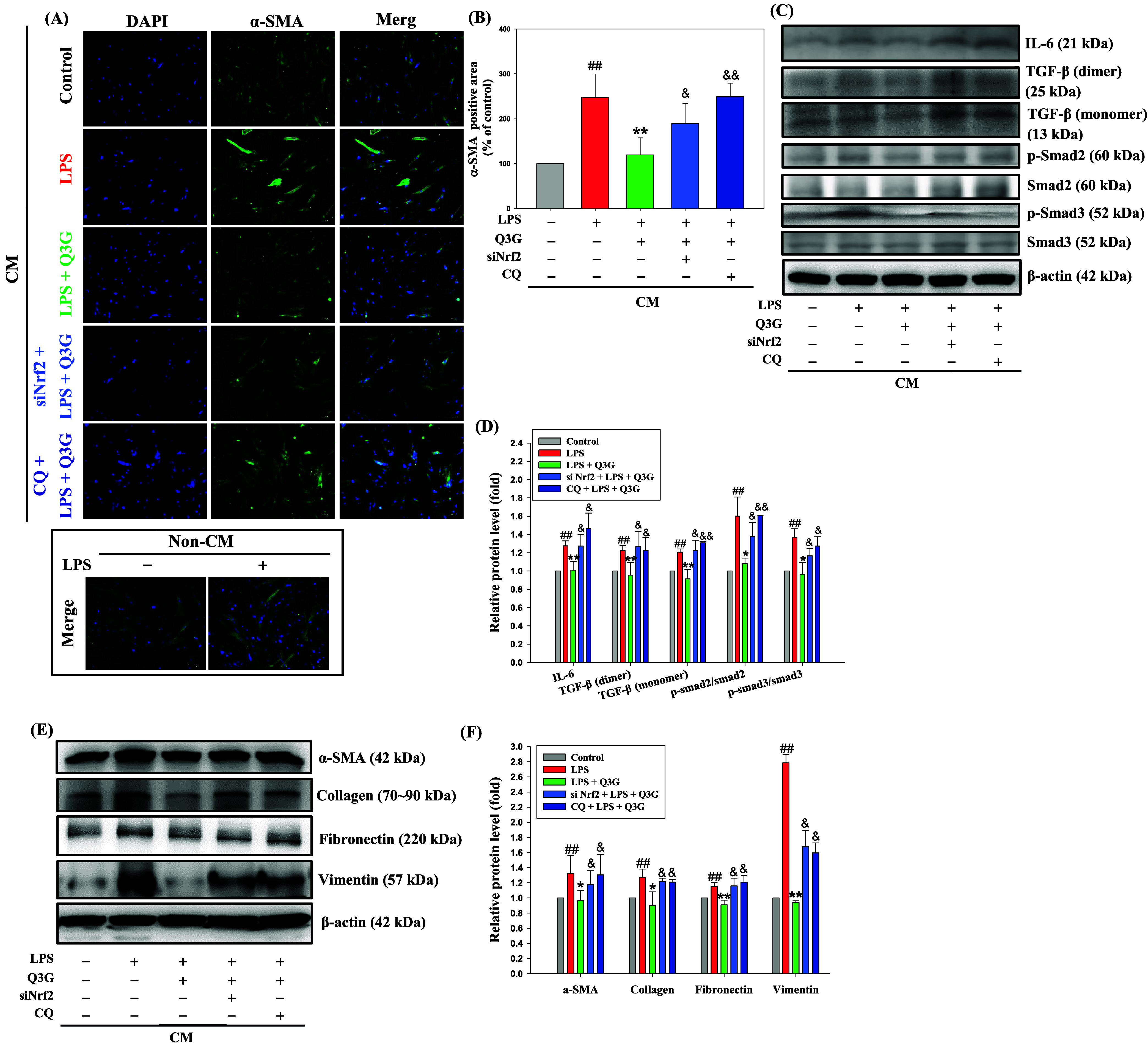
Nrf2 and autophagy are essential for the Q3G-declined FMT in the CM-induced MRC-5 cells. BEAS-2B cells were pretreated with or without Nrf2 siRNA for 12 h and CQ (3 μM) for 30 min, and then cotreated with Q3G (0.25 μM) for 6 h in the presence of LPS (10 μg/mL) for another 24 h. Then the conditioned medium was collected and treated MRC-5 cells for 72 h. (A) Photomicrographs of MRC-5 cells showed DAPI, α-SMA and merge staining (100×). The non-CM groups (control and LPS alone) were showed as compared group (*lower left panel*). (B) The α-SMA values were calculated for the α-SMA-positive area in each random field. The protein levels of IL-6, TGF-β, p-Smad2/Smad2, p-Smad3/Smad3 (C), and α-SMA, collagen, fibronectin and vimentin (E) were analyzed by Western blotting. β-actin was served as an internal control. (D and F) The quantitative data of protein levels are presented as mean ± SD (*n* ≥ 3) from at least three independent experiments. ^##^ *p* < 0.01 compared with the control group. *p < 0.05, **p < 0.01 compared with the LPS group. ^&^ *p* < 0.05 compared with the LPS plus Q3G group.

Furthermore, to elucidate the downstream signaling pathways involved in FMT, the activation of the TGF-β/Smad signaling axis was investigated. Western blot analysis showed that LPS-induced CM significantly triggered the phosphorylation of Smad2 and Smad3, as well as the upregulation of TGF-β. Treatment with Q3G effectively suppressed the phosphorylation of Smad2/3 and decreased TGF-β protein levels. These inhibitory effects of Q3G on Smad activation were reversed following Nrf2 knockdown or CQ treatment, as demonstrated in Figure 5C,5D. Consistent with the immunofluorescence results, Western blot analysis demonstrated elevated protein levels of fibrotic markers including α-SMA, collagen, fibronectin, and vimentin in the LPS-CM group (Figure 5E). Q3G markedly attenuated these increases, while this suppression was reversed by either Nrf2 silencing or CQ treatment. The expression levels of α-SMA, collagen, fibronectin, and vimentin are presented in Figure 5F. These findings suggested that Q3G inhibited epithelial–mesenchymal communication-mediated FMT in lung fibroblasts, and that this protective effect might be mediated by activation of Nrf2 and autophagy.

### 3.6 Effects of Q3G on Activation of Nrf2 and LC3-II in the CM-Induced MRC-5 Cells

Given the involvement of both Nrf2 signaling and autophagy in Q3G-mediated cytoprotection, the expression and localization of Nrf2 and LC3-II in CM-treated fibroblasts were further assessed. As shown in Figure 6A, LPS-induced CM slightly decreased the fluorescence signals of Nrf2 (red) and LC3-II (green) in MRC-5 cells relative to control. Q3G treatment markedly enhanced both signals, with evident cytoplasmic colocalization of Nrf2 and LC3-II. This effect was substantially diminished in cells transfected with Nrf2 siRNA or cotreated with CQ. Line profile analysis was conducted to assess the spatial colocalization of Nrf2 and LC3-II, revealing a pronounced overlap of their fluorescence signals along defined regions (red arrows). Quantitative analysis of the red/green fluorescence intensity ratio revealed a significant elevation in the Q3G group compared to the LPS group, which was notably reduced upon Nrf2 knockdown or CQ treatment (Figure 6B).

**Figure 6:**
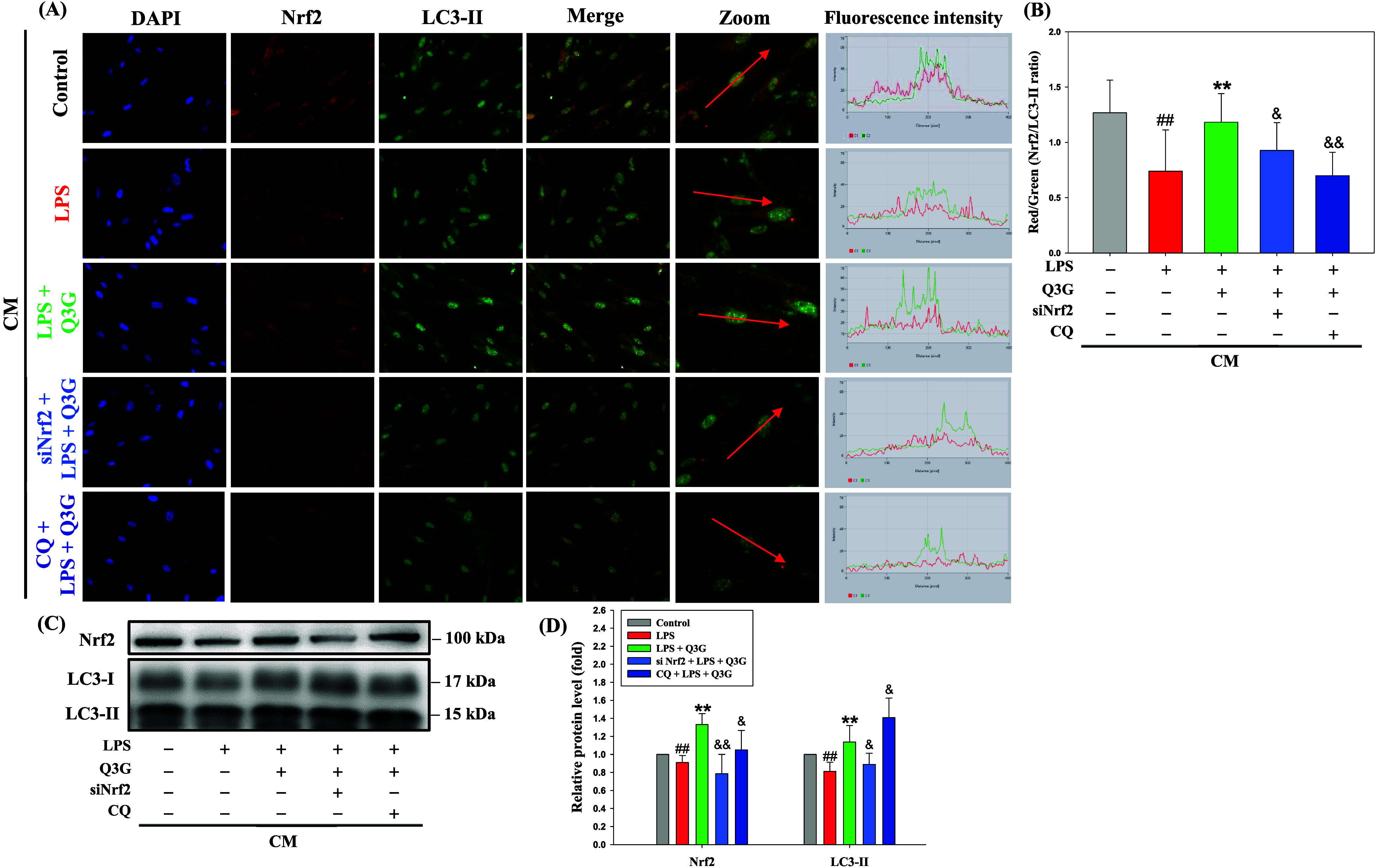
Effects of Q3G-mediated dual activation of Nrf2 and LC3-II in the CM-induced MRC-5 cells. BEAS-2B cells were pretreated with or without Nrf2 siRNA for 12 h and CQ (3 μM) for 30 min, and then cotreated with Q3G (0.25 μM) for 6 h in the presence of LPS (10 μg/mL) for another 24 h. Then the conditioned medium was collected and treated MRC-5 cells for 72 h. (A) Photomicrographs of MRC-5 cells showed DAPI, Nrf2, LC3-II, merge (200×), and zoom (400×) staining (*left panel*). The fluorescence intensity was identified by the red arrow and examined using ZEISS ZEN Microscopy Software (*right panel*). (B) The ratio of fluorescence intensity was calculated by Nrf2 (red) divided by LC3-II (green) in each random field. (C) The protein levels of Nrf2 and LC3-II were analyzed by Western blotting. β-actin was served as an internal control. (D) The quantitative data are presented as mean ± SD (*n* ≥ 3) from at least three independent experiments. ^##^ *p* < 0.01 compared with the control group. **p < 0.01 compared with the LPS group. ^&^ *p* < 0.05, ^&&^ *p* < 0.01 compared with the LPS plus Q3G group.

Furthermore, the nuclear translocation of Nrf2 was examined to assess its transcriptional activation. As shown in Figure S4A,B, LPS-induced CM suppressed the nuclear localization of Nrf2 in MRC-5 cells, whereas Q3G treatment significantly promoted its translocation into the nucleus. This Q3G-mediated nuclear accumulation was significantly attenuated by Nrf2 siRNA or CQ treatment. In parallel, Western blot analysis demonstrated that Q3G enhanced the expression of Nrf2 and LC3-II compared to the LPS group (Figure 6C). However, this upregulation was attenuated in the presence of Nrf2 silencing or CQ cotreatment. Notably, the accumulation of LC3-II observed under CQ treatment likely reflects the blockade of autophagosome–lysosome fusion, rather than increased autophagic flux, indicating a disrupted autophagy process (Figure 6D). These findings confirm that Q3G enhances Nrf2 and autophagy signaling in fibroblasts, and that both pathways are essential for its protective effects against CM-induced stress.

## 4 Discussion

COPD is a progressive lung disorder marked by persistent airway inflammation, irreversible airflow restriction, and structural alterations of the pulmonary architecture. Among its pathological subtypes, emphysema is characterized by the breakdown of alveolar walls and expansion of airspaces, resulting in compromised gas exchange and diminished lung elasticity. Notably, fibrotic remodeling is increasingly recognized in advanced COPD, contributing to small airway obstruction and disease progression.^5^ Currently, available antifibrotic agents such as pirfenidone and nintedanib are approved for pulmonary fibrosis; however, their clinical utility is constrained by several limitations, including modest efficacy, high cost, and undesirable side effects such as gastrointestinal disturbances, liver toxicity, and photosensitivity. Moreover, these agents primarily act to slow disease progression rather than offering curative potential. There is a pressing need for novel therapeutic compounds that can more effectively target the underlying molecular mechanisms of fibrotic lung diseases, particularly those involved in oxidative damage, impaired autophagy, and fibroblast-to-myofibroblast transition (FMT).^26^

The present study explored the therapeutic potential of Q3G in modulating key pathogenic pathways involved in fibrotic remodeling during chronic inflammatory lung injury. While our murine model recapitulated emphysematous features and exhibited hallmarks of fibrosis (Figure 1). In this context, the model effectively simulates fibrotic remodeling as a consequence of persistent inflammation and oxidative stress, which is increasingly recognized as a critical factor in small airway obstruction and advanced COPD progression. In the current study, we initially identified a notable reduction in the expression of Nrf2 and LC3-II in human lung tissue arrays from emphysema compared to normal lung, pointing toward impaired antioxidative defenses and dysregulated autophagy in COPD pathogenesis (Figure 1A–C). This observation is consistent with previous reports linking impaired Nrf2 signaling and autophagy dysfunction to COPD. Nrf2 is a transcription factor that regulates the expression of antioxidant enzymes, playing a pivotal role in protecting lung tissue from oxidative damage. Increasing evidence indicates that Nrf2 deficiency contributes to elevated oxidative stress and exacerbated pathological changes in COPD.^27^ In addition, dysregulation of autophagy may result in either insufficient clearance of damaged organelles or excessive degradation of essential cellular components, contributing to disease progression.^28^ Building upon these observations, a murine model of emphysema was established via a combination of LPS and elastase administration, which not only recapitulated the emphysematous features but also exhibited hallmarks of fibrosis, as demonstrated by collagen accumulation (Figure 1D,F). Importantly, the downregulation of Nrf2 and LC3-II noted in human tissues was likewise observed in the animal model (Figure 1D,E), suggesting a conserved mechanism linking oxidative imbalance and autophagy dysfunction with lung remodeling. Furthermore, the evidence of Figure 2(A,B) demonstrated the transition of lung fibroblasts into myofibroblasts in the LPS-challenged mice. Additional results confirmed that Q3G suppresses key fibrotic factors (TGF-β and collagen) associated with this transition (Figure 2C). Moreover, given that myofibroblasts can originate from multiple cell types,^29^ further investigations using lineage-tracing animal models will be essential to precisely characterize the specific cellular origins of FMT inhibition within the lung tissue. In the *in vitro* experiments, the results showed that Q3G not only upregulated the expression of Nrf2 and LC3-II, but also inhibited collagen production in MRC-5 lung fibroblasts and suppressed LPS-induced apoptotic factors (Figure 3). Additionally, previous study has shown that pulmonary fibrosis can be alleviated through autophagy-mediated activation of the Keap1/Nrf2 signaling pathway.^30^ The findings suggest Q3G’s potential role as a dual-pathway activator with antifibrotic effects.

Fibrosis is often observed as a late-stage consequence of chronic inflammation and tissue injury in COPD. Bronchial epithelial injury plays a critical role in initiating fibrotic processes by inducing oxidative stress and releasing pro-inflammatory cytokines, which in turn promote fibroblast activation and ECM deposition.^5^ To further investigate whether epithelial-derived factors contribute to fibroblast activation, a conditioned medium (CM) model was utilized, in which MRC-5 fibroblasts were exposed to medium collected from LPS-stimulated BEAS-2B bronchial epithelial cells (Figure 4A). The CM model employed in this study effectively mimicked epithelial–mesenchymal communication under inflammatory stress, a key mechanism implicated in fibrotic lung disease.^5^ Exposure of MRC-5 fibroblasts to CM derived from LPS-stimulated BEAS-2B cells significantly increased oxidative stress and the expression of pro-inflammatory cytokines (Figure 4), supporting the concept that epithelial injury contributes to fibroblast activation through paracrine signaling.^5^ Treatment with Q3G attenuated these effects by reducing intracellular ROS levels and suppressing inflammatory mediators, especially IL-6 (Figure 4C,D). IL-6 has been implicated in promoting chronic inflammation and fibrotic remodeling through the activation of downstream signaling pathways such as STAT3. IL-6 also drives fibroblast migration to injury sites and regulates their differentiation into myofibroblasts via the JAK/ERK pathway or through paracrine production of TGF-β at wound sites.^31^ In pulmonary fibrosis models, fibroblasts have been reported to secrete IL-6, leading to increased fibroblast proliferation and collagen synthesis.^32^ The marked inhibition of IL-6 by Q3G suggests that this compound may exert its protective effects not only by counteracting oxidative stress but also by modulating key inflammatory pathways involved in pulmonary injury and fibrosis. Consistently, Q3G attenuated FMT, as evidenced by reduced α-SMA expression and decreased levels of fibrotic proteins including collagen, fibronectin, and vimentin (Figure 5). Interestingly, although TGF-β expression did not show significant changes in the Inflammatory Cytokine Antibody Array (Figure 4D), Western blot analysis revealed a marked upregulation of TGF-β (Figure 5C), suggesting that Q3G may modulate TGF-β expression at the intracellular level, which may not be fully captured by secreted protein arrays. Notably, these effects were reversed upon Nrf2 silencing or cotreatment with the lysosomal inhibitor CQ. These findings highlight that Q3G disrupts key pathological cascades of pulmonary fibrosis, particularly oxidative stress, inflammation, and FMT, with its cytoprotective effects depending on both Nrf2 and autophagy pathways.

While our previous work established the antioxidant and anti-inflammatory roles of Q3G in acute lung injury by enhancing Nrf2 signaling and protective autophagy,^16^ the current study provides a significant conceptual advancement by focusing on fibrotic remodeling and FMT in a chronic context. A pivotal distinction of this work is the exploration of epithelial–mesenchymal communication. Unlike classical fibrosis models that focus primarily on fibroblast proliferation, our CM model allowed us to demonstrate that Q3G interrupts the pro-fibrotic crosstalk between injured epithelial cells and fibroblasts. Beyond individual cell protection, we demonstrated that Q3G disrupts the pro-fibrotic crosstalk between injured bronchial epithelial cells and adjacent fibroblasts. By utilizing a CM model, we provided evidence that Q3G modulates the secretory niche of epithelial cells, specifically reducing the release of pro-fibrotic mediators like TGF-β and IL-6 (Figure S2), thereby preventing the downstream activation of fibroblasts. Furthermore, this study identifies the canonical TGF-β/Smad2/3 signaling pathway as a critical downstream target of Q3G-mediated Nrf2 and autophagy activation (Figure 5C,D). Our data reveal a functional coupling between these two pathways; the pharmacological inhibition of autophagic flux by CQ effectively abolished Q3G-induced Nrf2 nuclear translocation and its subsequent suppression of the TGF-β/Smad signaling axis (Figures 5 and S4). This suggests that Q3G does not merely activate these pathways independently but relies on an intact autophagic process to facilitate Nrf2-mediated myofibroblast differentiation arrest. This mechanistic link provides a more comprehensive understanding of how Q3G exerts antifibrotic effects through a multicompartment regulatory approach.

Since BEAS-2B cells were stimulated with LPS, the potential confounding effect of residual LPS in the CM must be considered. However, this study provided several lines of evidence to distinguish the effects of epithelial secretory profiles from direct LPS exposure. Specifically, a non-CM control experiment demonstrated that direct treatment of MRC-5 cells with LPS failed to induce significant α-SMA expression, whereas the CM group showed a remarkable myofibroblast transition (Figure 5A). Furthermore, quantitative analysis confirmed that the CM was highly enriched with key pro-fibrotic cytokines, including TGF-β and IL-6 (Figure S2). While these data suggest that epithelial-derived cytokines are the primary drivers of the observed fibrotic changes, the possibility of synergistic effects between trace residual LPS and the cytokines cannot be entirely dismissed. Future research employing the Limulus Amebocyte Lysate (LAL) assay for LPS quantification or using specific neutralizing antibodies against TGF-β would be valuable to further define the precise contribution of each soluble factor.

Mounting evidence suggests a crosstalk between autophagy and Nrf2 signaling, in which autophagy may facilitate the degradation of Keap1, the cytoplasmic repressor of Nrf2, thereby enhancing Nrf2 activation.^30^ Conversely, Nrf2 can regulate autophagy-related genes, such as p62, under stress conditions. This reciprocal regulation offers a potential therapeutic axis for modulating fibrosis by restoring redox balance and promoting intracellular clearance mechanisms.^28^ In this study, the colocalization of Nrf2 and LC3-II in MRC-5 cells treated with CM suggests a potential interplay between oxidative stress regulation and autophagy activation. The increased overlap between Nrf2 and LC3-II observed in immunofluorescence analysis may reflect the recruitment of Nrf2 to autophagosomal structures or a shared signaling platform that coordinates cellular adaptation to stress (Figure 6). Together, these findings indicate that Q3G enhances both Nrf2 signaling and autophagy in fibroblasts exposed to CM, thereby attenuating oxidative stress and pro-inflammatory cytokine release. This dual activation contributes to the suppression of FMT and ECM accumulation, ultimately mitigating pulmonary fibrosis (Figure 7).

**Figure 7:**
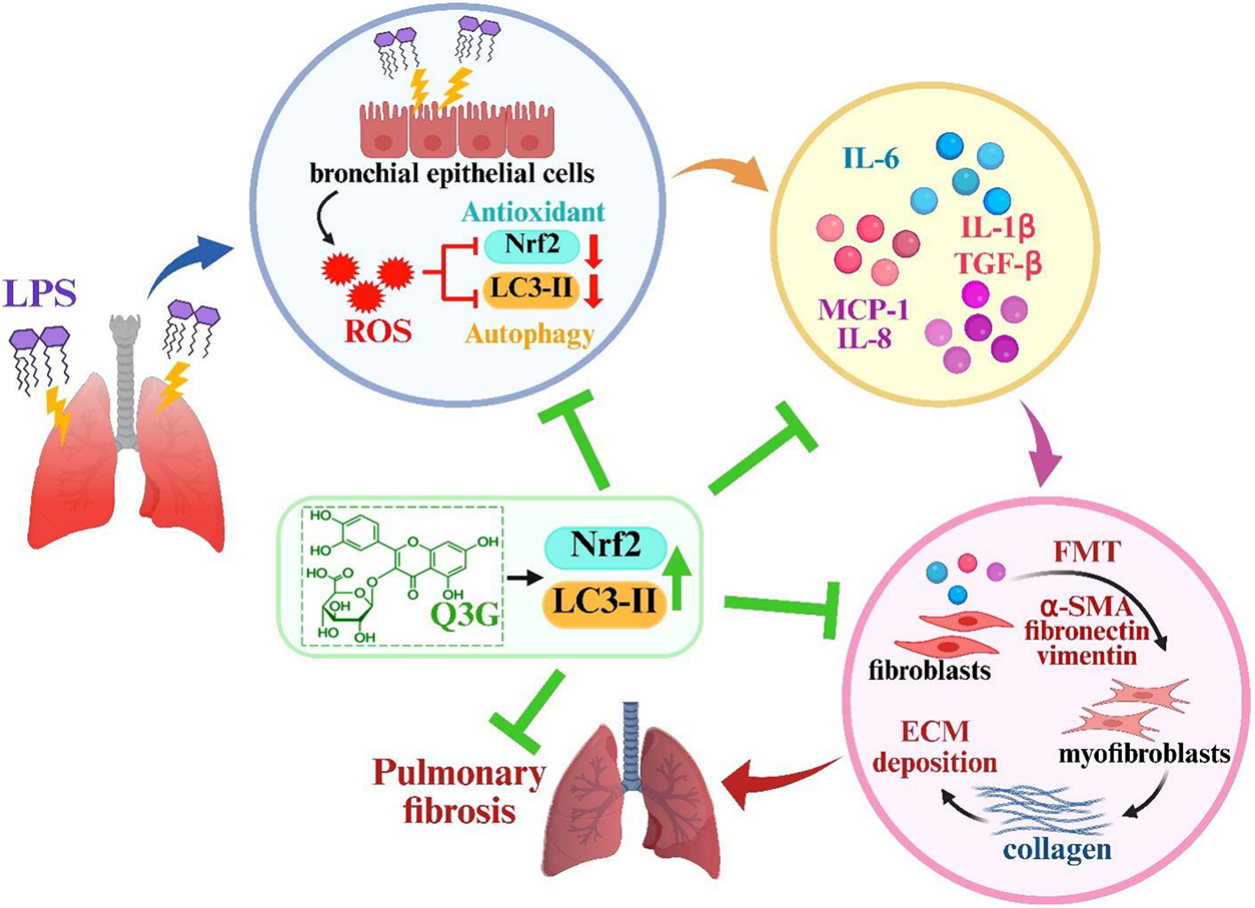
Overview of the Q3G-mediated antipulmonary fibrosis effect via dual activation of Nrf2 and autophagy. Q3G mediates the upregulation of Nrf2 and autophagy, which subsequently decrease the release of proinflammatory cytokines and FMT, leading to the improvement of pulmonary fibrosis. The schematic representation was created with BioRender.com.

While the present study provides compelling evidence for the dual role of Q3G in activating Nrf2 signaling and autophagy to mitigate pulmonary fibrosis, further research is warranted to elucidate the precise molecular mechanisms underlying these effects. In particular, the involvement of the p62/Keap1 axis in mediating the crosstalk between autophagy and Nrf2 activation should be explored, as this pathway may serve as a critical regulatory node in the cellular response to oxidative stress and fibrotic stimuli.^33^ Additionally, comprehensive transcriptomic or proteomic analyses could provide deeper insights into the global gene and protein expression changes induced by Q3G, revealing additional therapeutic targets or pathways beyond Nrf2 and autophagy. Moreover, emerging evidence suggests that Nrf2 and autophagy are intricately linked with inflammatory pathways.^34^ Nrf2 activation has been shown to suppress IL-6 expression by modulating oxidative stress and inhibiting NF-κB activity,^35^ while autophagy can control cytokine production through degradation of inflammasome components.^36^ Given the observed inhibition of IL-6 by Q3G alongside enhanced Nrf2 and autophagy activity, future studies should investigate whether Q3G directly modulates IL-6 expression through these pathways or if IL-6 reduction is a secondary effect. Clarifying the mechanistic interplay among Nrf2, autophagy, and IL-6 will provide deeper insight into the anti-inflammatory and antifibrotic properties of Q3G. Employing Nrf2- or autophagy-deficient models, as well as targeted modulation of IL-6 signaling, could further delineate these complex relationships. Collectively, these future directions aim to validate and expand the therapeutic potential of Q3G as a novel agent targeting the multifaceted pathogenesis of pulmonary fibrosis.

## 5 Limitation

While our study establishes that Q3G-mediated protection requires the functional coupling of Nrf2 and autophagy, the precise molecular trigger initiating this axis remains to be elucidated. Future temporal kinetic analyses will be necessary to delineate the exact hierarchical relationship between these pathways. In addition, although pharmacological inhibition (CQ) and siRNA silencing yielded consistent functional evidence, we recognize that chemical inhibitors may exert off-target effects. Future studies employing transgenic models, such as Nrf2-knockout or autophagy-deficient systems, would be instrumental to validate our findings in a systemic physiological context and rule out nonspecific effects. Lastly, regarding the Q3G dosage, while the concentration used (0.15 μmol/mouse) is physiologically relevant and established as nontoxic,^15,37^ detailed data on its tissue-specific pharmacokinetics remains limited. Future investigations focusing on the bioavailability and metabolic kinetics of Q3G in lung tissues are warranted to provide the pharmacological depth required to fully realize its clinical translational value in pulmonary fibrosis.

## 6 Conclusion

In summary, this study demonstrates that Q3G exerts protective effects against fibrotic remodeling driven by epithelial–mesenchymal crosstalk in chronic lung injury by targeting two key cellular defense mechanisms: the activation of the Nrf2 antioxidant pathway and the enhancement of autophagy. A conditioned medium model was employed to mimic fibroblast activation in response to epithelial injury. Under these conditions, Q3G significantly attenuated oxidative stress, pro-inflammatory cytokine production, and fibroblast-to-myofibroblast transition. The inhibitory effects of Q3G on ROS generation, cytokine secretion, and fibrotic marker expression were markedly diminished following Nrf2 knockdown or autophagy inhibition, thereby emphasizing the critical roles of these pathways. Furthermore, the observed colocalization of Nrf2 and LC3-II suggests a mechanistic interaction underlying the dual regulatory effects of Q3G (Figure 7). Collectively, these findings highlight the potential of Q3G in mitigating fibrotic remodeling associated with chronic inflammation and support its development as a therapeutic candidate for chronic pulmonary fibrotic disorders.

## Supplementary Material



## Data Availability

All data of this study are included in this article.

## Notes

Effects of Q3G on Nrf2, LC3-I/II, and IL-1β protein levels in LPS-induced BEAS-2B cells (Figure S1); results of ELISAs and a heatmap for various pro-inflammatory and pro-fibrotic cytokines (TGF-β, IL-1β, IL-6, and TNF-α) in conditioned medium (Figure S2); and cell viability data (Figure S3) along with Nrf2 nuclear translocation analysis (Figure S4) in CM-treated MRC-5 cells (PDF)

## Abbreviations

FMT: fibroblast-to-myofibroblast transition
Q3G: quercetin-3-glucuronide
Nrf2: nuclear factor-erythroid 2 related factor 2
ROS: reactive oxygen species
IL: interleukin
ECM: extracellular matrix
COPD: chronic obstructive pulmonary disease
LPS: lipopolysaccharide
TNF-α: tumor necrosis factor-α
TGF-β: transforming growth factor-β
α-SMA: α-smooth muscle actin
LC3: microtubule-associated protein 1 light chain 3
CM: conditioned medium
CQ: chloroquine
