# Supplementary material for: Dietary Quercetin-3-Glucuronide Mitigates Oxidative Stress, Inflammation, and Fibroblast Transition by Regulating Nrf2 and Autophagy in Pulmonary Fibrosis
Source: J Agric Food Chem. 2026 Jan 14;74(21):16334–48. doi: 10.1021/acs.jafc.5c13444 (PMC13237778; doi:10.1021/acs.jafc.5c13444)
Supplement: Supplementary file 1 [file jf5c13444_si_001.pdf]

Supporting Information

Dietary Quercetin-3-Glucuronide Mitigates Oxidative Stress, Inflammation, and Fibroblast Transition by Regulating Nrf2 and Autophagy in Pulmonary Fibrosis

Pei-Rong Yu[a], Chiao-Yun Tseng[a], Yu-Hsuan Liang[a], Yu-Ci Chang[b],

Jing-Hsien Chen[a,c,1] and Hui-Hsuan Lin[b,c,*,1]

[a] Department of Nutrition, Chung Shan Medical University, Taichung City 40201, Taiwan

[b]Department of Medical Laboratory and Biotechnology, Chung Shan Medical University, Taichung City 40201, Taiwan

[c] Clinical Laboratory, Chung Shan Medical University Hospital, Taichung City 40201, Taiwan

[*] Correspondence author: Hui-Hsuan Lin, Department of Medical Laboratory and Biotechnology, Chung Shan Medical University, No. 110, Sec. 1, Jianguo N. Road, Taichung City 40201, Taiwan. Tel: (886) 4-24730022, ext. 12410. Fax: (886) 4-23248171. E-mail: linhh@csmu.edu.tw.

[1] These authors contributed equally to this work and therefore share corresponding authorship.

Fig. S1

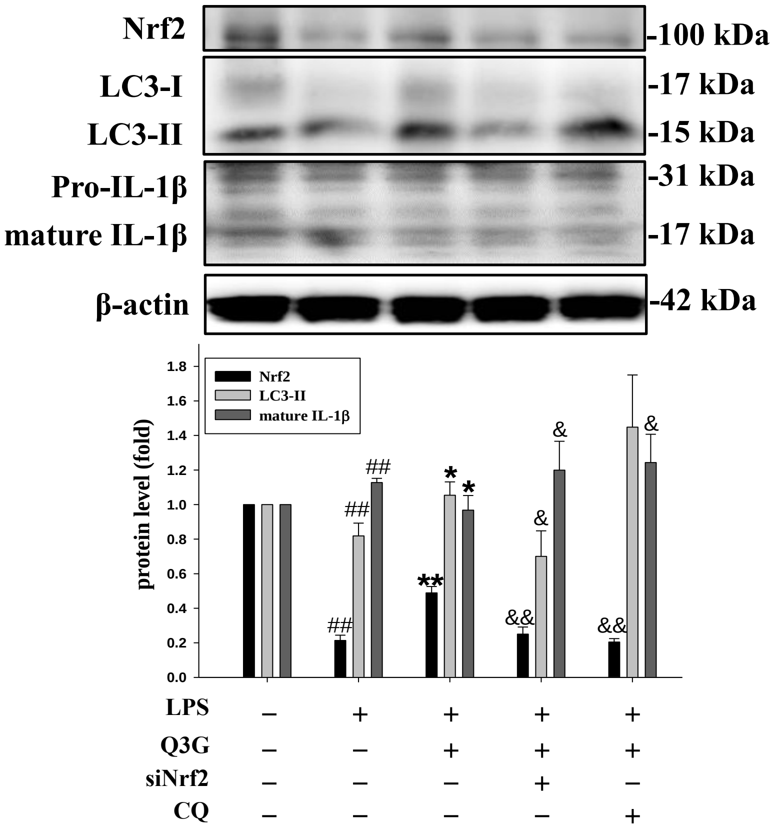

Fig. S1. Effects of Q3G on Nrf2, LC3I/II and IL-1β in the LPS-induced BEAS-2B cells. BEAS-2B cells were pre-treated with or without Nrf2 siRNA for 12 h and CQ (3 μM) for 30 min, and then co-treated with Q3G (0.25 μM) for 6 h in the presence of LPS (10 μg/mL) for another 24 h. The protein levels of Nrf2, LC3-I/II, and IL-1β were analyzed by Western blotting. β-actin was served as an internal control. The quantitative data are presented as mean ± SD ($n \geq 3$) from at least three independent experiments. $^{\#\#}p< 0.01$ compared with the control group. $^{*}p< 0.05$, $^{**}p< 0.01$ compared with the LPS group. $^{\&}p< 0.05$, $^{\&\&}p< 0.01$ compared with the LPS plus Q3G group.

Fig. S2

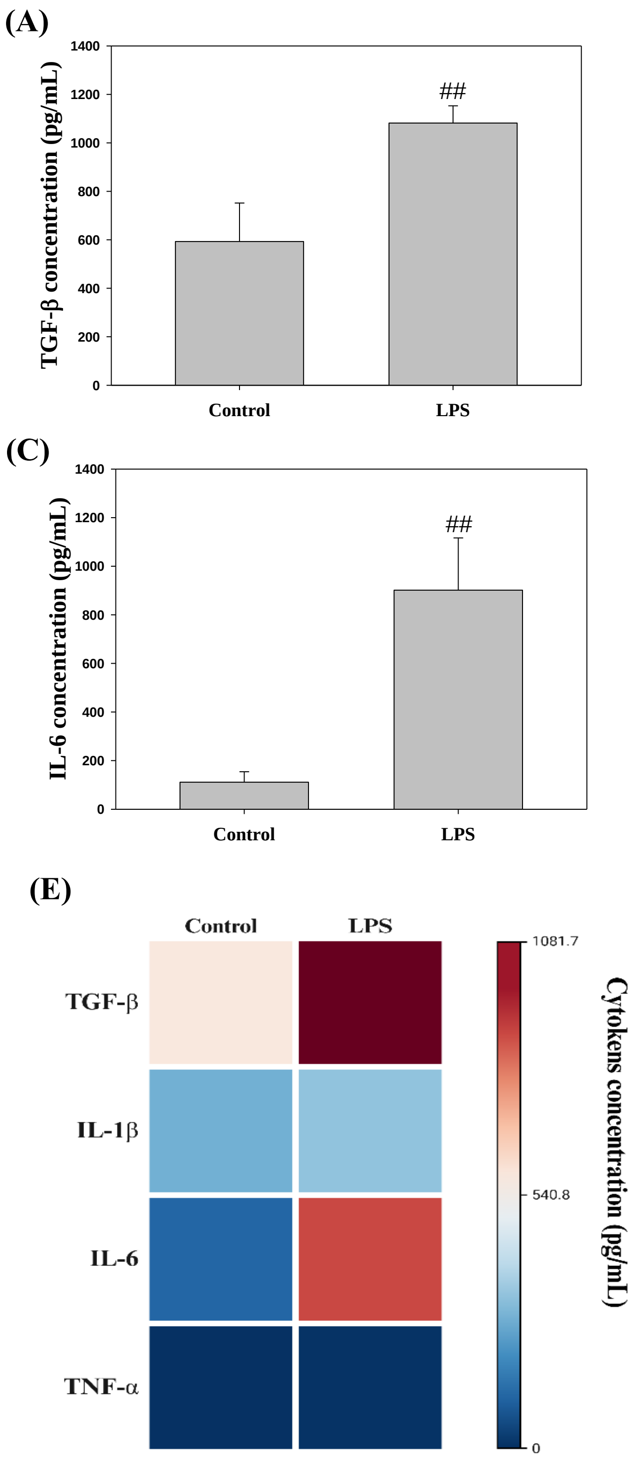

Fig. S2. The presences of key pro-inflammatory and pro-fibrotic cytokines in the CM derived from LPS-treated BEAS-2B cells. BEAS-2B cells were treated with or without LPS (10 μg/mL) for 24 h. Then the conditioned medium was collected and detected the levels of TGF-β **(A)**, IL-1β **(B)**, IL-6 **(C)**, and TNF-α **(D)** by ELISAs. The quantitative data are presented as mean ± SD ($n \geq 3$) from at least three independent experiments. $^{\#}p< 0.05$, $^{\#\#}p< 0.01$ compared with the control group. **(E)** The levels of cytokines, including TGF-β, IL-1β, IL-6, and TNF-α, are presented as a heatmap from high (red color) to low (blue color).

Fig. S3

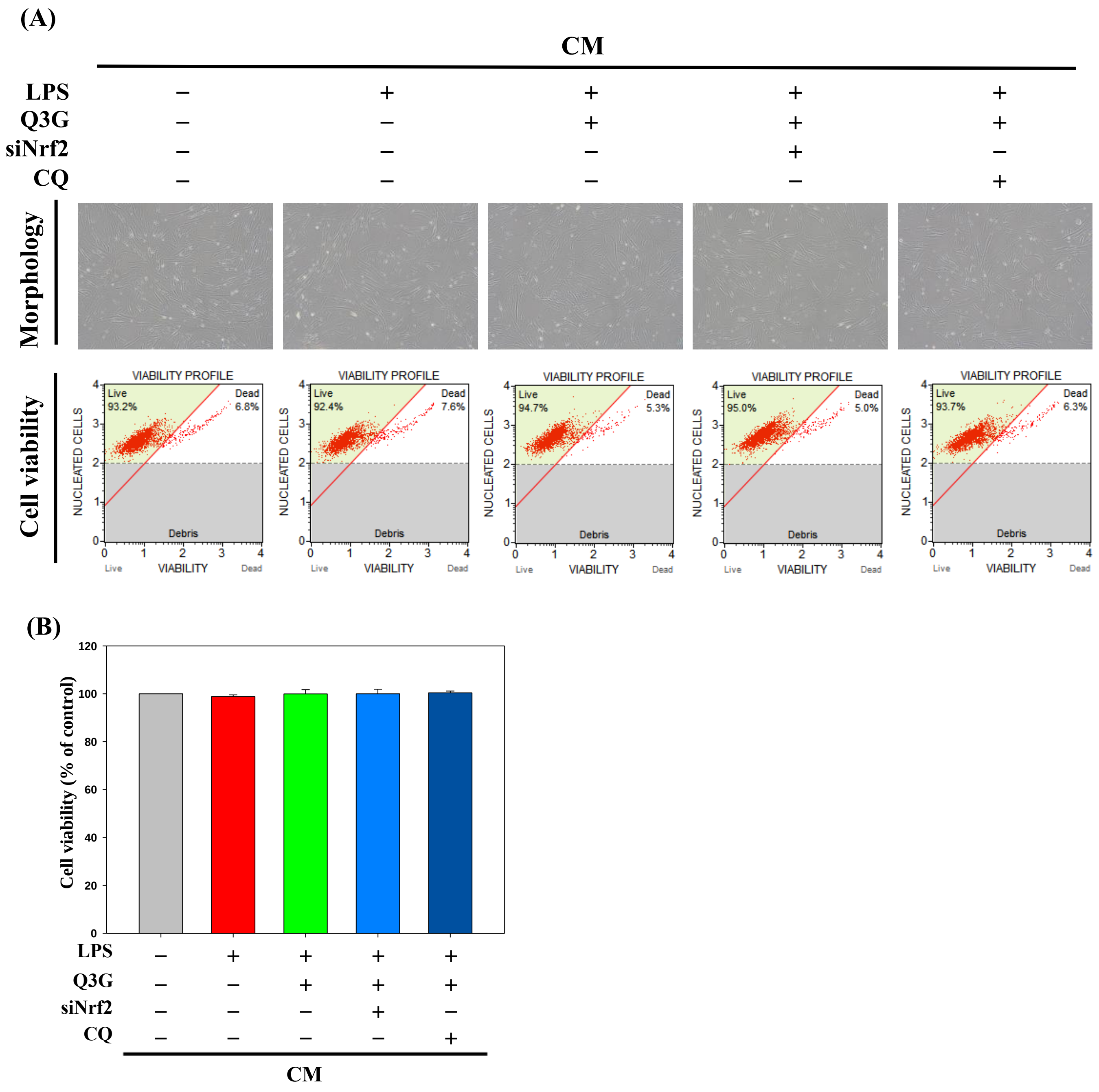

Fig. S3. Effects of Q3G on cell viability in the CM-induced MRC-5 cells. BEAS-2B cells were pre-treated with or without Nrf2 siRNA for 12 h and CQ (3 μM) for 30 min, and then co-treated with Q3G (0.25 μM) for 6 h in the presence of LPS (10 μg/mL) for another 24 h. Then the conditioned medium (CM) was collected and treated MRC-5 cells for 72 h. **(A)** Photomicrographs of MRC-5 cells in CM treatment (100x, *upper panel*). The cell viability was measured by Muse™ Cell Analyzer (*lower panel*). **(B)** The quantitative data of cell viability are presented as mean ± SD (n=3) from three independent experiments.

Fig. S4

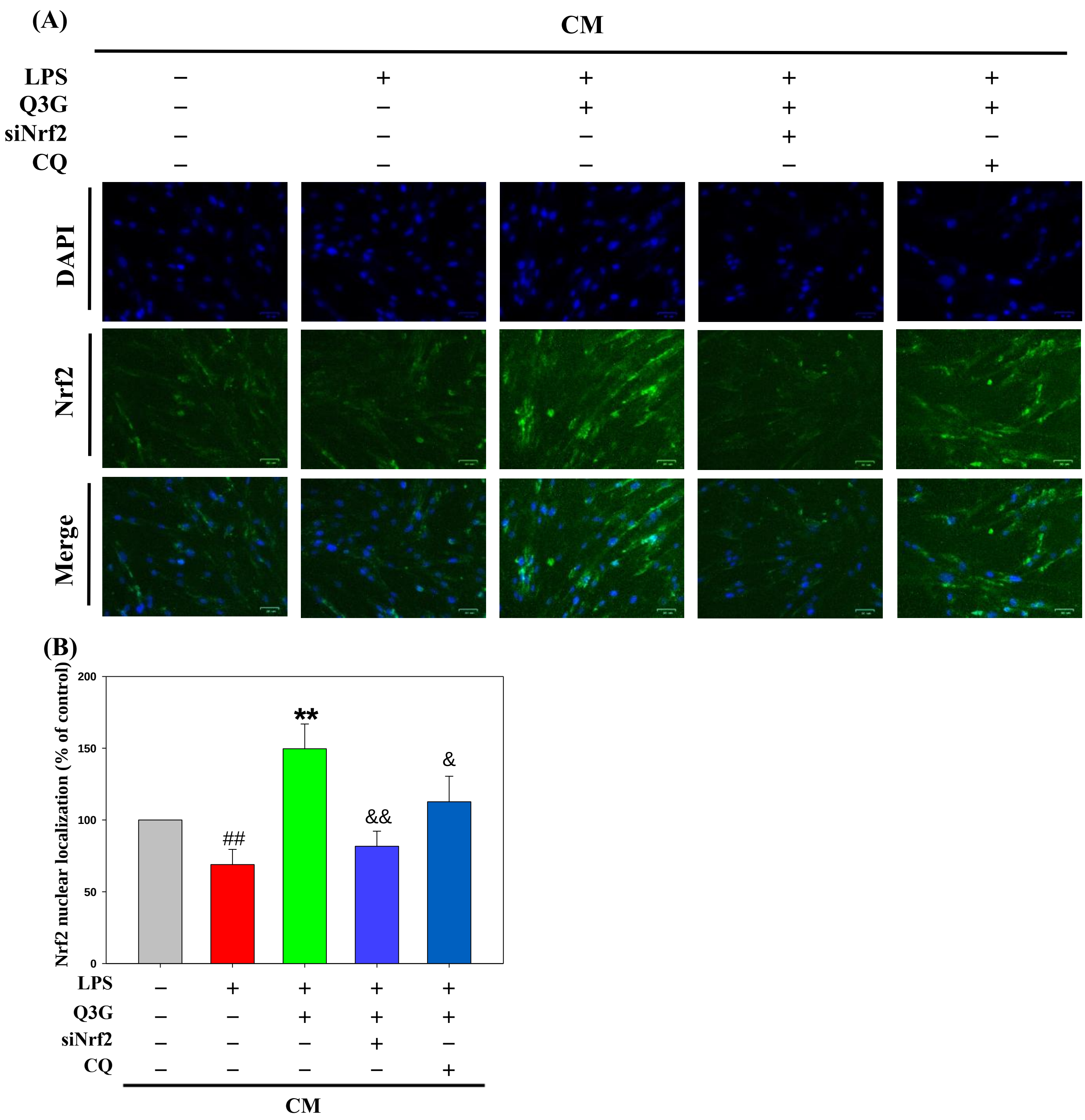

Fig. S4. Effects of Q3G on Nrf2 nuclear translocation in the CM-induced MRC-5 cells. BEAS-2B cells were pre-treated with or without Nrf2 siRNA for 12 h and CQ (3 μM) for 30 min, and then co-treated with Q3G (0.25 μM) for 6 h in the presence of LPS (10 μg/mL) for another 24 h. Then the conditioned medium (CM) was collected and treated MRC-5 cells for 72 h. **(A)** Photomicrographs of MRC-5 cells showed DAPI, Nrf2 and merge staining (200x). **(B)** The quantitative data of Nrf2 nuclear localization are presented as mean ± SD (n=3) from three independent experiments. The quantitative data are presented as mean ± SD (n ≥ 3) from at least three independent experiments. $^{\#\#}p < 0.01$ compared with the control group. $^{**}p < 0.01$ compared with the LPS group. $^{\&}p < 0.05$, $^{\&\&}p < 0.01$ compared with the LPS plus Q3G group.
